# The Potential of Radiomics Nomogram in Non-invasively Prediction of Epidermal Growth Factor Receptor Mutation Status and Subtypes in Lung Adenocarcinoma

**DOI:** 10.3389/fonc.2019.01485

**Published:** 2020-01-09

**Authors:** Wei Zhao, Yuzhi Wu, Ya'nan Xu, Yingli Sun, Pan Gao, Mingyu Tan, Weiling Ma, Cheng Li, Liang Jin, Yanqing Hua, Jun Liu, Ming Li

**Affiliations:** ^1^Department of Radiology, Second Xiangya Hospital, Central South University, Changsha, China; ^2^Department of Radiology, Huadong Hospital Affiliated to Fudan University, Shanghai, China; ^3^School of Biomedical Engineering, Capital Medical University, Beijing, China; ^4^Diagnosis and Treatment Center of Small Lung Nodules of Huadong Hospital, Shanghai, China; ^5^Institute of Functional and Molecular Medical Imaging, Fudan University, Shanghai, China

**Keywords:** *EGFR*, radiomics, nomogram, lung adenocarcinomas, CT

## Abstract

**Purpose:** Up to 50% of Asian patients with NSCLC have *EGFR* gene mutations, indicating that selecting eligible patients for *EGFR*-TKIs treatments is clinically important. The aim of the study is to develop and validate radiomics-based nomograms, integrating radiomics, CT features and clinical characteristics, to non-invasively predict *EGFR* mutation status and subtypes.

**Materials and Methods:** We included 637 patients with lung adenocarcinomas, who performed the *EGFR* mutations analysis in the current study. The whole dataset was randomly split into a training dataset (*n* = 322) and validation dataset (*n* = 315). A sub-dataset of *EGFR*-mutant lesions (*EGFR* mutation in exon 19 and in exon 21) was used to explore the capability of radiomic features for predicting *EGFR* mutation subtypes. Four hundred seventy-five radiomic features were extracted and a radiomics sore (R-score) was constructed by using the least absolute shrinkage and selection operator (LASSO) regression in the training dataset. A radiomics-based nomogram, incorporating clinical characteristics, CT features and R-score was developed in the training dataset and evaluated in the validation dataset.

**Results:** The constructed R-scores achieved promising performance on predicting *EGFR* mutation status and subtypes, with AUCs of 0.694 and 0.708 in two validation datasets, respectively. Moreover, the constructed radiomics-based nomograms excelled the R-scores, clinical, CT features alone in terms of predicting *EGFR* mutation status and subtypes, with AUCs of 0.734 and 0.757 in two validation datasets, respectively.

**Conclusions:** Radiomics-based nomogram, incorporating clinical characteristics, CT features and radiomic features, can non-invasively and efficiently predict the *EGFR* mutation status and thus potentially fulfill the ultimate purpose of precision medicine. The methodology is a possible promising strategy to predict *EGFR* mutation subtypes, providing the support of clinical treatment scenario.

## Key points

1. We developed and validated two Radiomics-based nomograms, incorporating clinical characteristics, CT features and radiomic features, to non-invasively predict the *EGFR* mutation status and subtypes with the aim to potentially fulfill the ultimate purpose of precision medicine.
2. The presented results indicate that radiomics-based nomogram may potentially facilitate scalable precision medicine on identifying eligible patients of lung adenocarcinoma for EGFR-targeted therapy.

## Introduction

Lung cancer is the leading cause cancer-related death both in male and female (1). Non-small cell lung cancer (NSCLC) accounts for more than 80% of lung cancers, of which lung adenocarcinoma is the most common histological subtype (2). With the advances of genomics, molecular-targeted therapy like using tyrosine kinase inhibitors (TKIs), which targets the epidermal growth factor receptor (*EGFR*) mutations, is recommended as first-line system therapy before first-line therapy by National Comprehensive Cancer Network (NCCN) for patients with advanced *EGFR*-mutant NSCLC (2) and proved to substantially improve the progression-free survival (PFS) compared with conventional chemotherapy (3, 4). Up to 50% of Asian patients with NSCLC have *EGFR* gene mutations (5), indicating that selecting eligible patients for EGFR-TKIs treatments is clinically important. In patients with NSCLC, the most commonly found *EGFR* mutations are deletions in exon 19 (45%) and in exon 21 (L858R in 40%) in patients with *EGFR* mutations (2). Both mutations are associated with sensitivity to the small molecule TKIs as well as erlotinib, gefitinib, afatinib, and osimertinib (2), however, with different survival outcomes in response to both *EGFR*-TKIs and chemotherapy (6). Therefore, identifying *EGFR* mutation subtypes, especially those responsive to TKI treatment, seems to be more critically and scientifically important than just predicting *EGFR* mutation status.

In this context, though more and more research has emerged on the non-invasive prediction of EGFR mutation status in recent years (7–9), no predictors are recommended for selecting patients in clinical decision-making. Moreover, substantial discrepancies are presented to date with regarding to some features, especially semantic features derived from medical images (10). Buoyed by the availability of big data and state-of-art data analysis strategy, such as radiomics and deep learning, decoding tumor phenotype to precisely predict genotype has becoming the point of attention (11). Several studies have investigated the potential ability of radiomics to non-invasively predicting *EGFR* mutation status and show promising results (12–15). Few results are finally applied in clinical practice yet due to the complicated procedure (e.g., time consuming, poor reproducibility, remaining the operator-dependency that is not biases-free, and so on) of radiomic researches (16). In view of this, models that giving an individual numerical probability of a clinical event (e.g., nomogram) rather than a predicting accuracy, may be more suitable and convenient for clinical application.

In the current study, we aim to build radiomics-based nomograms, integrating radiomics, CT imaging features and clinical characteristics, to non-invasively predict *EGFR* mutation status and subtypes (exon 19 and 21mutation).

## Materials and Methods

### Patient Selection and Dataset Preparation

**Figure 1:**
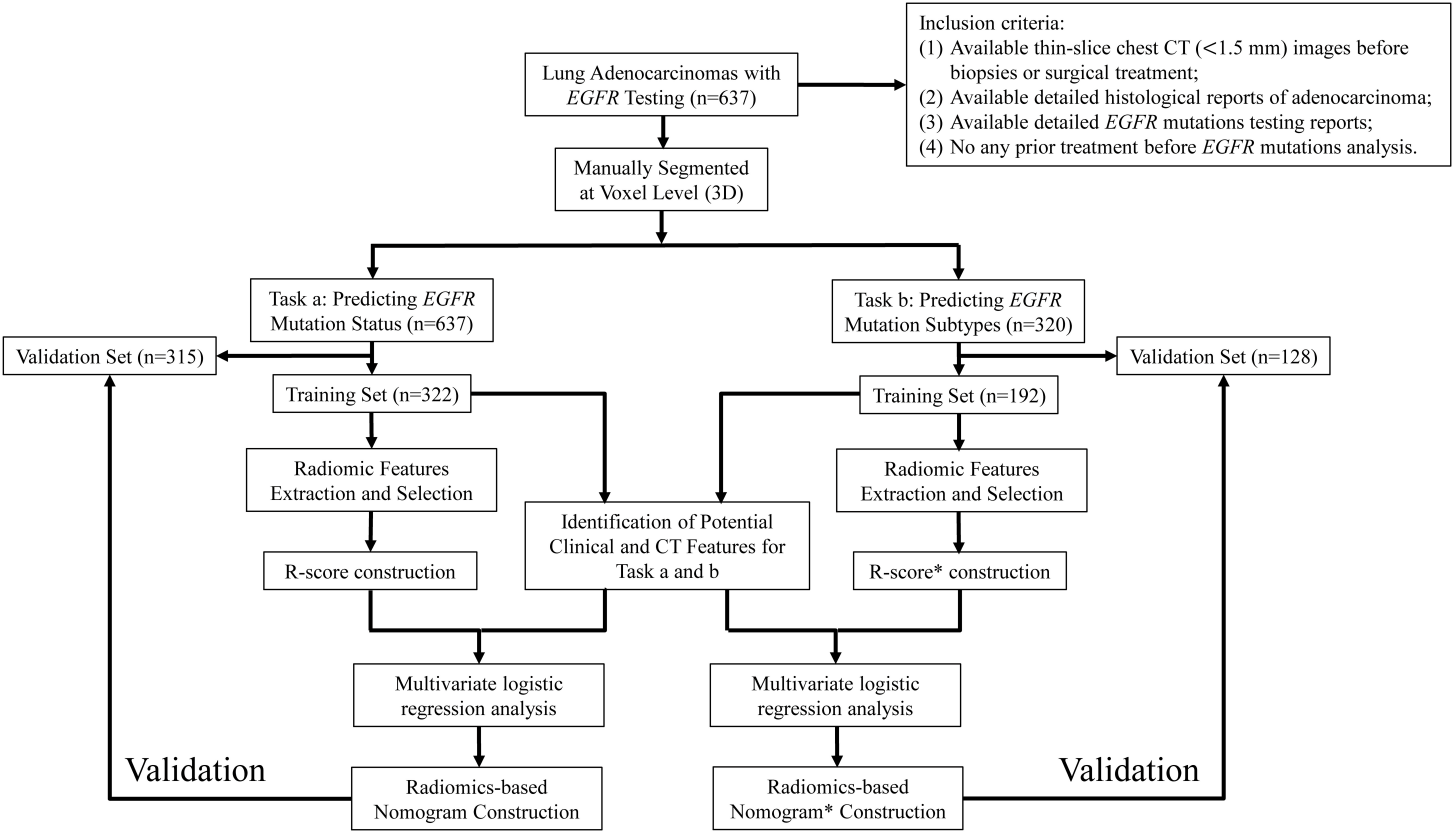
The flowchart of the study.

This retrospective study was approved by the institutional review board (No. 20170103), which waived the requirement for patients' informed consent referring to the CIOMS guideline. The flowchart of our study was showed in Figure 1.

A search of Picture Achieving and Communication System (PACS) and pathological system from January 2013 to June 2018 was performed by one author with the following inclusion criteria: (1) Available thin-slice chest CT (<1.5 mm) images before biopsies or surgical treatment; (2) Available detailed histological reports of adenocarcinoma; (3) Available detailed *EGFR* mutations testing reports; (4) No any prior treatment before *EGFR* mutations analysis.

Finally, 637 patients were included. Of the 637 lesions, 342 lesions tested positively for an *EGFR* mutation (*EGFR* Mut), where 295 lesions were classified as wild-type lung adenocarcinomas (*EGFR* WT). Note that only one malignant nodule was studied for each patient due to the availability of *EGFR* testing report. Among the 342 patients with *EGFR* Mut, 130 patients were detected an *EGFR* Mut in exon 19, whereas 190 patients were detected an *EGFR* Mut in exon 21. The clinical and histopathologic variables, including age, sex, smoking status, tumor size, tumor location, histological subtypes etc. were presented in Table 1.

**Table 1:** Clinical and histological characteristics of included patients.

**Characteristics**	**Number**	**Percentage**
Gender		
Male	269	42.2
Female	368	57.8
Mean age (range) (year)		
Male	62.0 ± 11.8 (27–85)	–
Female	58.3 ± 11.9 (22–85)	–
Total	59.9 ± 12.0 (22–85)	–
Mean size (range) (cm)	1.85 ± 1.29 (0.4–8.6)	–
Smoke		
Never smoker	588	92.3
Current or former smoker	49	7.7
Location		
Right lobe	378	59.3
Left lobe	259	40.7
Pathology		
Adenocarcinoma *in situ*	32	5.0
Minimally invasive adenocarcinoma	174	27.3
Invasive adenocarcinoma	431	67.7
TMN classification (eighth edition)		
0	32	5.0
I	388	60.9
II	10	1.6
III	12	1.9
IV	195	30.6
*EGFR* Mut	342	53.4
*EGFR* Mut in exon 19	130	38.0
*EGFR* Mut in exon 21	190	55.6

Two tasks were investigated in the current study: task (a), differentiating *EGFR* Mut from *EGFR* WT; task (b), differentiating *EGFR* Mut in exon 19 from in exon 21. Each dataset in two tasks (*n* = 637 and *n* = 320) was split into 10 groups (1–10), each subset was randomly selected by choosing 10% of each of the 2 categories. In task (a), groups 1–5 were defined as training dataset, the rest groups were defined as validation dataset. In task (b), considering the insufficient training data, groups 1–6 were defined as training dataset, the rest groups were defined as validation dataset. Note that we only included *EGFR* mutations in exon 19 and 21 in task b to avoid the sparse training data and the disbalance of data distribution. Mover, constructing a model to predict these two exons in patients with EGFR mutation is clinically reasonable (see Introduction).

### CT Acquisition and Imaging Interpretation

All included patients were performed with the following six scanners: GE Discovery CT750 HD, 64-slice LightSpeed VCT, Revolution CT (GE Medical Systems); Somatom Definition flash, Somatom Sensation-16, Somatom Force (Siemens Medical Solutions). The acquisition parameters were as follows: 120 kVp; 100– 200 mAs; pitch, 0.75–1.5; and collimation, 1–1.5 mm, respectively. All imaging data were reconstructed by using a medium sharp reconstruction algorithm with a thickness of 0.75–1.5 mm. CT images were acquired in the supine position at full inspiration for all patients. Only plain CT images were used in the current study. Two radiologists (with 12 and 3 years of experience in chest CT interpretation) independently interpreted the CT images, blinded to clinical and histologic findings. Thirteen CT features (Table 1) were evaluated. The definitions of these features were described in previous study (17, 18). A re-evaluation for achieving a consensus was performed to solve the disagreement between two radiologists.

### Segmentation and Radiomic Features Extraction

All nodules were manually delineated slice by slice using a medical image processing and navigation software 3D Slicer (version 4.8.0, Brigham and Women's Hospital) by one author (with 5 years of experience in chest CT interpretation), then the volume of interests (VOIs) were confirmed by another radiologist (with 12 years of experience in chest CT interpretation). Fifty randomly selected nodules were independently segmented by the two authors for feature reproductive analysis. Images and VOIs with NII format were exported for further analysis. Radiomic features from three categories, including 50 gray-level histogram features, 325 gray-level co-occurrence matrix (GLCM) features, and 100 gray-level run lengths matrix (GLRLM) features, were extracted using Matlab 2016b (MathWorks, Natick, USA). The details of extracted radiomic features were presented in Supplementary Data. Radiomic feature extraction methodology was described in our previous study (18).

### Features Selection and Radiomic Score Construction

The least absolute shrinkage and selection operator (LASSO) method, which is an accepted algorithm for feature selection in high-dimensional variables (19), was applied to select the features that were most distinguishable and build a logistic regression model in training dataset. Then a radiomic score (R-score) was calculated for each lesion using features selected by LASSO and weighted by the respective coefficients.

### Nomogram Construction and Validation

Univariate analysis was firstly to identify the potential predictors among clinical characteristics, CT features and R-score. Factors that associated with *EGFR* mutation status and subtypes were then included to multivariate analysis to identify the independent predictors. Furthermore, the identified independent factors were selected to construct the final nomogram in the training dataset.

### Histologic Evaluation and EGFR Mutation Analysis

The included lung adenocarcinomas were categorized according to the 2011 IASLC/ATS/ERS classification system (20) (drug target-associated). Molecular analysis of mutation status of EGFR exons 18–21 was examined using a PCR-based amplification-refractory mutation system (ARMS) by AmoyDx company.

### Statistical Analysis

Statistical analysis was performed using R software (version 3.4.2; http://www.Rproject.org). The mean values and standard deviations were expressed for continuous variables (age, lesions size), and frequency or percentage for categorical variables. The Wilcoxon rank sum test and the *x*^2^-test were used to compare medians and proportions between two groups, respectively. Predictive performance was evaluated by the area under the curve (AUC) of the receiver operator characteristic (ROC). DeLong test was used to evaluate the difference of the ROCs between various models (21). Hosmer-Lemeshow test was used to evaluate the goodness-of-fit of the constructed nomogram. ICC analysis was performed using “irr” package, features with an ICC> 075 were consider as robust features. Lasso logistic regression was done using the “glmnet” package. Multivariate logistic regression, nomograms and calibration plots were done with the “rms” package. A *P*<0.05 indicated a significant difference.

## Results

### Associations Between Clinical, CT Features and EGFR Mutation Status and Subtypes

Associations between clinical, CT features and *EGFR* mutation status and subtypes were presented in Tables 2, 3. The incidence of harboring *EGFR* mutation was significantly higher in female than male in two datasets (*P* = 0.002, *P* = 0.013, respectively). Patients with *EGFR* Mut had a higher age in the current study. Smoking was not a significant factor to differentiate *EGFR* Mut lesions from *EGFR* WT lesions. In terms of radiographic features, 9 features, including size, margin, shape, pleural retraction, bronchiole change, lobulation, speculation, peripheral emphysema, peripheral fibrosis were significantly associated with *EGFR* mutation status. Patients with *EGFR* mutations in 21 exon had a higher age than those with *EGFR* mutations in 19 exon in two datasets (*P* = 0.013, *P* = 0.003, respectively). No other clinical and CT features were identified as potential factors to predict the *EGFR* mutation subtypes.

**Table 2:** Basal characteristics of patients in training and validation set (task a).

**Characteristics**	**Training set (*****n*** **= 322)**		***P***	**Validation set (*****n*** **= 315)**		***P***
	***EGFR* Mut (*n* = 172)**	***EGFR* WT (*n* = 150)**		***EGFR* Mut (*n* = 170)**	***EGFR* WT (*n* = 145)**	
**Clinical Characteristics**						
Gender			0.002			0.013
Male	50 (29.1)	69 (46)		70 (41.2)	80 (55.2)	
Female	122 (70.9)	81 (54)		100 (58.8)	65 (44.8)	
Age (year)	61.92 ± 57.64	57.46 ± 12.59	0.001	61.85 ± 11.63	57.63 ± 11.73	0.000
Smoke			0.154			0.404
Never smoker	163 (94.8)	136 (90.7)		158 (92.9)	131 (90.3)	
Current or former smoker	9 (5.2)	14 (9.3)		12 (7.1)	14 (9.7)	
**Radiographic Characteristics**						
Size (cm)	2.09 ± 1.24	1.53 ± 1.26	0.000	2.06 ± 1.31	1.65 ± 1.25	0.000
Type			0.091			0.472
Pure or part solid GGN	117 (68.0)	103 (68.7)		111 (65.3)	89 (61.4)	
Solid	55 (32.0)	47 (31.3)		59 (34.7)	56 (38.6)	
Margin			0.002			0.039
Easily differentiated	52 (30.2)	71 (47.3)		58 (34.1)	66 (45.5)	
Uneasily differentiated	120 (69.8)	79 (52.7)		112 (65.9)	79 (54.5)	
Shape			0.000			0.007
Round or oval	41 (23.8)	67 (44.7)		46 (27.1)	60 (41.4)	
Irregular	131 (76.2)	83 (55.3)		124 (72.9)	85 (58.6)	
Pleural retraction			0.000			0.015
Present	87 (50.6)	46 (30.7)		83 (48.8)	51 (35.2)	
Absent	85 (49.3)	104 (69.3)		87 (51.2)	94 (64.8)	
Bubble lucency			0.317			0.978
Present	51 (29.7)	37 (24.7)		49 (28.8)	42 (29.0)	
Absent	121 (70.3)	113 (75.3)		121 (71.2)	103 (71.0)	
Vascular change			0.050			0.575
Present	120 (69.8)	89 (59.3)		113 (66.5)	92 (63.4)	
Absent	52 (30.2)	61 (40.7)		57 (33.5)	53 (36.6)	
Bronchiole change			0.000			0.003
Present	90 (52.3)	44 (29.3)		86 (50.6)	49 (33.8)	
Absent	82 (47.7)	106 (70.7)		84 (49.4)	96 (66.2)	
Lobulation			0.031			0.034
Present	79 (45.9)	87 (58.0)		70 (41.2)	77 (53.1)	
Absent	93 (54.7)	63 (42.0)		100 (58.8)	68 (46.9)	
Spiculation			0.003			0.041
Present	87 (50.6)	51 (34.0)		80 (47.1)	85 (58.6)	
Absent	85 (49.4)	99 (66.0)		90 (52.9)	60 (41.4)	
Peripheral Emphysema			0.014			0.002
Present	3 (1.7)	11 (7.3)		6 (3.5)	19 (13.1)	
Absent	169 (98.3)	139 (92.7)		164 (96.5)	126 (86.9)	
Peripheral fibrosis			0.022			0.009
Present	55 (32.0)	31 (20.7)		63 (37.1)	34 (23.4)	
Absent	117 (68.0)	119 (79.3)		107 (62.9)	111 (76.6)	
Pleural effusion			0.177			0.506
Present	6 (3.5)	1 (0.7)		1 (0.6)	3 (2.1)	
Absent	166 (96.5)	149 (99.3)		169 (99.4)	142 (97.9)	
R-score	−0.40 ± 0.50	0.05 ± 0.68	0.000	−0.37 ± 0.51	0.01 ± 0.0.58	0.000

**Table 3:** Basal characteristics of patients in training and validation set (task b).

**Characteristics**	**Training set (*****n*** **= 192)**		***P***	**Validation set (*****n*** **= 128)**		***P***
	***EGFR* Mut in exon 19 (*n* = 78)**	***EGFR* Mut in exon 21 (*n* = 114)**		***EGFR* Mut in exon 19 (*n* = 52)**	***EGFR* Mut in exon 21 (*n* = 76)**	
**Clinical Characteristics**						
Gender			0.848			0.858
Male	27 (34.6)	41 (36.0)		17 (32.7)	26 (34.2)	
Female	51 (65.4)	73 (64.0)		35 (67.3)	50 (65.8)	
Age (year)	59.82 ± 11.66	64.13 ± 10.47	0.013	57.77 ± 12.90	64.70 ± 11.13	0.003
Smoke			0.595			0.853
Never smoker	74 (94.9)	106 (93.0)		49 (94.2)	71 (93.4)	
Current or former smoker	4 (5.1)	8 (7.0)		3 (5.8)	5 (6.6)	
**Radiographic Characteristics**						
Size (cm)	2.07 ± 1.30	2.19 ± 1.27	0.432	1.77 ± 0.98	2.26 ± 1.39	0.051
Type			0.264			0.458
Pure or part solid GGN	48 (61.5)	79 (69.3)		33 (63.5)	53 (69.7)	
Solid	30 (38.5)	35 (30.7)		19 (36.5)	23 (30.3)	
Margin			0.412			0.477
Easily differentiated	21 (26.9)	37 (32.5)		16 (30.8)	28 (36.8)	
Uneasily differentiated	57 (73.1)	77 (67.5)		36 (69.2)	48 (63.2)	
Shape			0.869			0.064
Round or oval	17 (21.8)	26 (22.8)		17 (32.7)	14 (18.4)	
Irregular	61 (78.2)	88 (77.2)		35 (67.3)	62 (81.6)	
Pleural retraction			0.344			0.620
Present	41 (52.6)	52 (45.6)		29 (55.8)	39 (51.3)	
Absent	37 (47.4)	62 (54.4)		23 (44.2)	37 (48.7)	
Bubble lucency			0.567			0.895
Present	19 (24.4)	32 (28.1)		17 (32.7)	24 (31.6)	
Absent	59 (75.6)	82 (71.9)		35 (67.3)	52 (68.4)	
Vascular change			0.663			0.497
Present	55 (70.5)	77 (67.5)		34 (65.4)	54 (71.1)	
Absent	23 (29.5)	37 (32.5)		18 (34.6)	22 (28.9)	
Bronchiole Change			0.739			0.284
Present	45 (57.7)	63 (55.3)		21 (40.4)	38 (50.0)	
Absent	33 (42.3)	51 (44.7)		31 (59.6)	38 (50.0)	
Lobulation			0.113			0.831
Present	52 (66.7)	63 (55.3)		27 (51.9)	38 (50.0)	
Absent	26 (33.3)	51 (44.7)		25 (48.1)	38 (50.0)	
Spiculation			0.179			0.566
Present	46 (59.0)	56 (49.1)		28 (53.8)	37 (48.7)	
Absent	32 (41.0)	58 (50.9)		24 (46.2)	39 (51.3)	
Peripheral emphysema			0.394			0.698
Present	0 (0.0)	3 (2.6)		2 (3.8)	2 (2.6)	
Absent	78 (100.0)	111 (97.4)		50 (96.2)	74 (97.4)	
Peripheral fibrosis			0.673			0.216
Present	29 (37.2)	39 (34.2)		15 (28.8)	30 (39.5)	
Absent	49 (62.8)	75 (65.8)		37 (71.2)	46 (60.5)	
Pleural effusion			0.898			0.795
Present	1 (1.3)	3 (2.6)		1 (1.9)	2 (2.6)	
Absent	77 (98.7)	111 (97.4)		51 (98.1)	74 (97.4)	
R-score^*^	−0.39 ± 1.35	0.27 ± 1.12	0.000	−0.70 ± 1.25	0.23 ± 1.12	0.000

### Construction of R-Score and the Association Between R-Score (R-Score^*^) and EGFR Mutation Status and Subtypes

**Figure 2:**
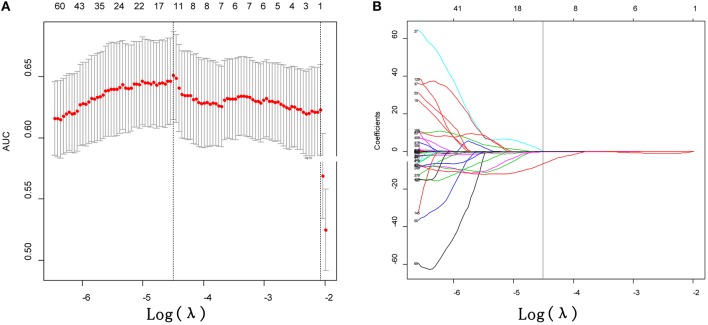
Feature selection using the least absolute shrinkage and selection operator (LASSO) binary logistic regression model. **(A)** Tuning parameter (λ) selection in the LASSO model used 10-fold cross-validation via minimum criteria. The area under the receiver operating characteristic (AUC) curve was plotted vs. log (λ). Dotted vertical lines were drawn at the optimal values by using the minimum criteria. **(B)** LASSO coefficient profiles of the 425 texture features. A coefficient profile plot was produced against the log (λ) sequence. Vertical line was drawn at the value selected using 10-fold cross-validation, where optimal λ (−4.497) resulted in 11 non-zero coefficients.

After performing ICC analysis, 425 of 475 radiomics were identified as robust features. The LASSO logistic regression model was performed to select the most distinguishable features in training dataset, resulting in 11 features left (Figure 2). Subsequently, the 11 potential predictors were consequently conducted into a R-score by using the following formula: R-score = −1.072477 + 0.007008 ^*^ mean_10_0 + 0.038891 ^*^ Homogeneity 0_90_0 + 1.86E-05 ^*^ Contrast 45_45_0 + 8.54E-05 ^*^ Contrast 90_135_0 + 6.29E-05 ^*^ Contrast 90_135_1 – 0.039584 ^*^ skewness_1.5 – 0.254939 ^*^ skewness_2 + 1.15E-06 ^*^ RLN_90_2.5 + 7.46E-05 ^*^ Contrast 90_90_2.5 – 9.69E-05 ^*^ Contrast 0_0_2.5 – 6.342383311 ^*^ Homogeneity 0_0_2.5. The formula caption was presented in Supplementary Data (Referring to the formula for calculating R-score^*^).

**Figure 3:**
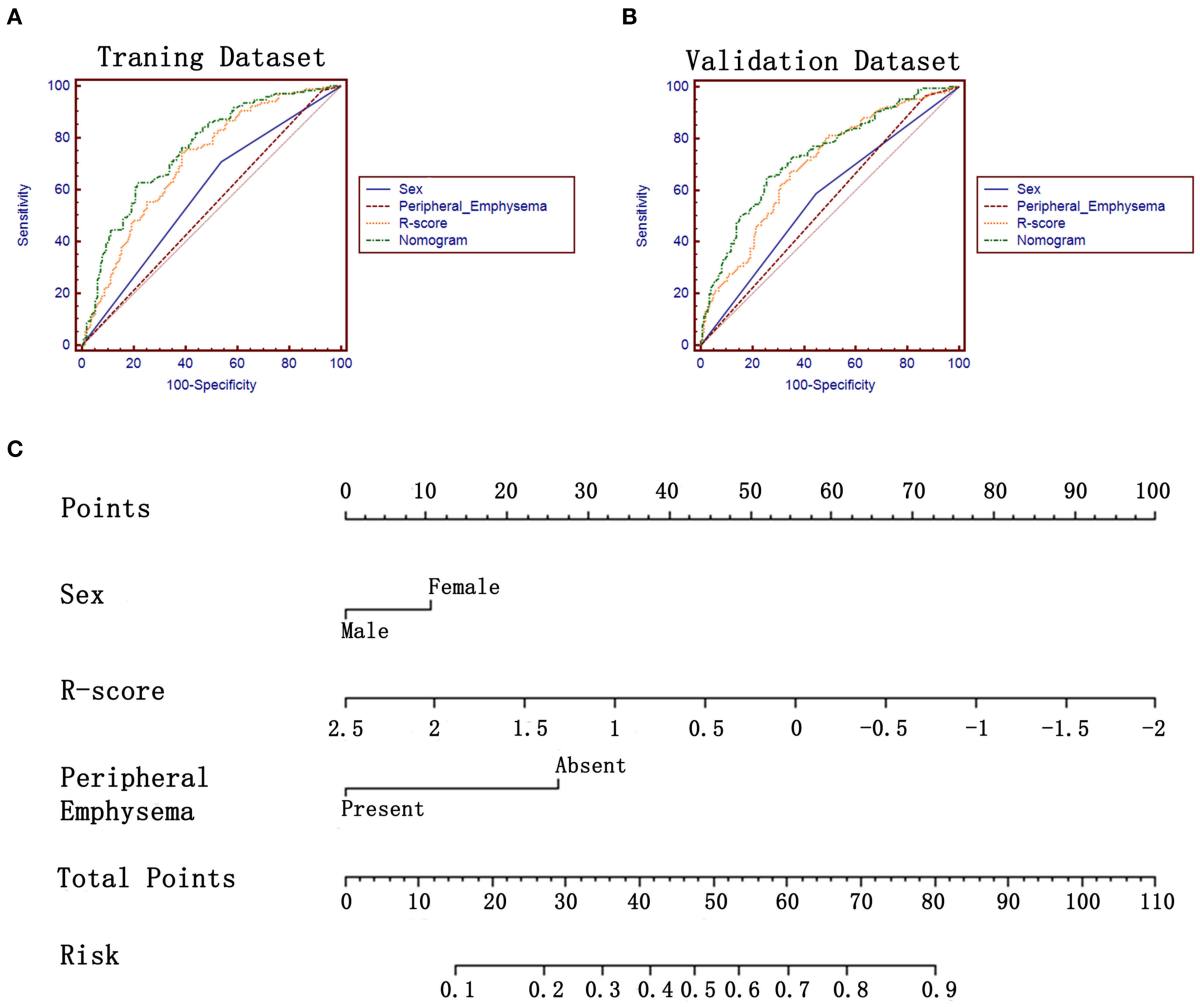
ROC analysis and the constructed nomogram in task a. **(A,B)** ROC analysis of sex, R-score, peripheral emphysema and the constructed nomogram in training and validation datasets, respectively. **(C)** The constructed nomogram for predicting *EGFR* mutation status.

The R-score was calculated for each lesion in two datasets of task a. *EGFR*-Mut lung adenocarcinomas had a lower R-score than *EGFR*-WT ones in training dataset (−0.40 ± 0.50 vs. 0.05 ± 0.68, *P* = 0.000), which was confirmed in validation dataset (−0.37 ± 0.51 vs. 0.01 ± 0.0.58, *P* = 0.000) (Table 2). The proposed R-score showed a good performance in differentiating *EGFR* mutation status with AUCs of 0.708, 0.694 in training and validation datasets (Figures 3A,B). The Hosmer-Lemeshow test for R-score yielded a non-significant statistic in the training and validation datasets (*P* = 0.644, *P* = 0.657, respectively), indicating that there was no departure from a perfect fit.

**Figure 4:**
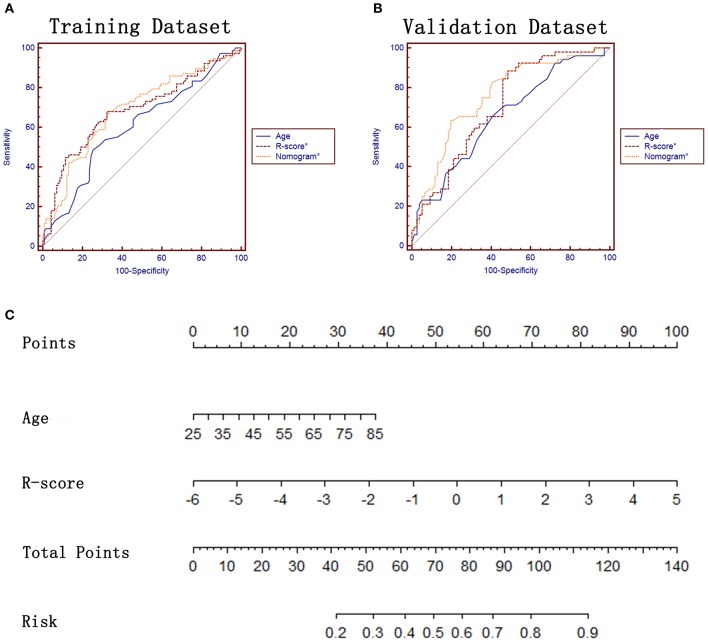
ROC analysis and the constructed nomogram in task b. **(A,B)** ROC analysis of age, R-score* and the constructed nomogram* in training and validation datasets, respectively. **(C)** The constructed nomogram* for predicting *EGFR* mutation subtypes.

In terms of the task of predicting the *EGFR* mutation subtypes (task b), 32 features were finally left after performing the LASSO analysis (Supplementary Data). A R-score^*^ was also calculated for each lesion by using the formula presented in the Supplementary Data. Lung adenocarcinomas with *EGFR* Mut in exon 19 had a lower R-score^*^ than ones with *EGFR* Mut in exon 21 in training dataset (−0.39 ± 1.35 vs. 0.27 ± 1.12, *P* = 0.000) and in validation dataset (−0.70 ± 1.25 vs. 0.23 ± 1.12, *P* = 0.000) (Table 3). The proposed R-score^*^ demonstrated a good performance in differentiating *EGFR* mutation subtypes with AUCs of 0.684, 0.708 in two datasets (Figures 4A,B). The Hosmer-Lemeshow test for R-score^*^ yielded a non-significant statistic in the training and validation set (*P* = 0.295, *P* = 0.242, respectively), which suggested that there was no departure from a perfect fit.

### Development and Validation of the Radiomics Nomograms for Predicting the EGFR Mutation Status and Subtypes

There was no multicollinearity between the significant factors identified by univariate analysis and R-score. After performing the multivariate analysis, sex, peripheral emphysema and R-score were identified as independent prognostic factors of harboring *EGFR* mutation (Table 4) and subsequently incorporated to develop the radiomics-based nomogram (Figure 3C). The constructed nomogram obtained a significantly incremental performance for predicting *EGFR* mutation status compared with that of sex and peripheral emphysema (Table 5). The Hosmer-Lemeshow test for the nomogram yielded a non-significant statistic in the training and validation set (*P* = 0.313, *P* = 0.816, respectively), which suggested that there was no departure from a perfect fit. Though the nomogram significantly outperformed R-score in training dataset, the statistically difference was not found in validation dataset (Table 5).

**Table 4:** Multivariate logistic regression of risk characteristics for predicting the *EGFR* mutation status and subtypes in training datasets.

**Characteristic**	**OR (95% CI)**	***P***
**Task a**		
Sex	2.291 (1.329–3.949)	0.003
Peripheral emphysema	5.412 (1.292–22.669)	0.021
R-score	2.262 (1.660–4.415)	0.000
Age	0.988 (0.965–1.012)	0.329
Size	1.58 (0.768–1.458)	0.729
Margin	1.362 (0.769–2.413)	0.290
Shape	1.343 (0.742–2.431)	0.330
Pleural retraction	0.702 (0.392–1.257)	0.234
Bronchiole change	0.661 (0.328–1.329)	0.245
Lobulation	1.278 (0.726–2.250)	0.395
Spiculation	0.953 (0.489–1.860)	0.888
Peripheral fibrosis	1.166 (0.561–2.240)	0.681
**Task b**		
Age	1.588 (1.041–2.421)	0.032
R-score^*^	1.921 (1.283–2.875)	0.002

*OR, odds ratio; CI, confidence interval; NA, not available*.

**Table 5:** Predictive performance of risk factors in two datasets regarding two tasks.

**Characteristic**	**AUC**	
	**Training dataset**	**Validation dataset**
**Task a**		
Sex	0.585^#^	0.570^#^
Peripheral Emphysema	0.528^#^	0.548^#^
R-score	0.708^#^	0.694
Nomogram	0.755	0.734
**Task b**		
Age	0.605^#^	0.656^#^
R-score^*^	0.684	0.708
Nomogram^*^	0.689	0.757

* *The constructed R-score and nomogram in task b*.

# *Significantly difference between the performance of nomogram and other factors*.

In terms of task b, age and R-score^*^ were identified as independent prognostic factors of predicting *EGFR* mutation subtypes (Table 4) and subsequently incorporated to develop the radiomics-based nomogram^*^ (Figure 4C). The Hosmer-Lemeshow test for the nomogram^*^ yielded a non-significant statistic in the training and validation set (*P* = 0.760, *P* = 0.413, respectively), indicating that there was no departure from a perfect fit. Not surprisingly, the constructed nomogram^*^ significantly outperformed age and potentially outperformed R-score^*^ in predicting *EGFR* mutation subtypes (Table 5).

## Discussion

Non-invasively and preoperatively predicting the *EGFR* mutation status, a field attracted continuous efforts of researchers, can overcome the disadvantages of molecular assays (e.g., high cost, intratumoral heterogeneity, poor sample quality) well and furtherly help clinicians to select the eligible patients for targeted therapy. Moreover, attempting to predict the *EGFR* mutation subtypes, especially those are sensitivity to small TKIs, may provide important information for making finer treatment scenario. In the current study, we developed and validated two radiomics based nomograms, incorporating clinical characteristics, CT features and radiomics, to predict the *EGFR* mutation status and subtypes with promising performance (AUC = 0.734, AUC = 0.757, respectively).

It is well-documented that the *EGFR* Mut rate are significantly higher in female than male (22), which is also confirmed in our study (*P* = 0.002 and *P* = 0.013 in two datasets, respectively). Note that smoking status, another potential clinical factor verified by previous studies (8, 14, 17), failed to show significant association with *EGFR* mutation status in the current study. Note that smoking status is still a contentious risk factor (23) and not recommended to use as the criteria for selecting eligible patients (2). Another reason why the difference between *EGFR* Mut and WT in terms of smoking status is diminished may be that the incidence of lung adenocarcinomas in female was higher than male (20, 24). Moreover, female may be less likely to be current/former smoker than male. Wu et al. (25) had reported that younger patients (<50 years old) with lung adenocarcinoma had lower EGFR mutation rate, which was not verified in multivariate logistic analysis in the current study. The discrepancy may be contributed to the data bias (ours vs. Wu's: 20.9 vs. 15.5%).

Many prior studies have investigated the role of morphological features in predicting *EGFR* mutation status. The results remain controversial. On one hand, most radiographic features are non-quantitative subjective features and susceptible to the discrepancy of evaluation caused by different knowledge structure of observers. On the other hand, obvious radiographical features are more frequently presented in advanced tumors instead of early-stage tumors. For example, stage I lung cancers account for 60–70% of detected lung cancers in screening programs (26). In our study, 60.9% of included patients were stage I lung cancers. In this context, the differentiating performance of these semantic features may be compromised. Peripheral emphysema was the only independent risk factors for predicting *EGFR* mutation status in the current study.

Another disadvantage of semantic features is that they only reflect few tumor information in biological level. By contrast, radiomics method can encode a more comprehensive level of feature abstraction and thus potentially provide better prediction performance. Previous studies have revealed the potential associations between these engineered features and *EGFR* mutation status (12, 23, 27) and proved that the performance of models can benefit from the integration of radiomics and clinical features (14, 23). These results were also confirmed in our study. Despite the promising results, the complicated process of feature extraction and the inconvenience of formula-based model limit its application in clinical context. Hence, an easy-to-use way for radiomics method is urgently needed. Incorporating multiple radiomic features into a radiomics score can tactfully make multi-marker analyses less complicated to use (18, 28), which is similar to the construction of multi-factor panels. In this study, the LASSO regression model was used to select and compress the radiomic features and thus construct the R-score. The constructed R-score outperformed the clinical and CT features in predicting *EGFR* mutation status (Table 5). Another strategy to make radiomics method friendly use is presenting the results in a more intuitive way, such as the nomogram (29, 30). Nomogram is a statistic model that can provide an individual numerical probability of a clinical event by integrating multiple variables (31, 32). To comprehensively investigate the predictive performance of non-invasively available variables, including clinical factors, imaging semantic features, imaging radiomic features, we adopted the two above-mentioned strategies to construct a radiomics-based nomogram for predicting the *EGFR* mutation status. The constructed nomogram in the current study was conveniently used to individualized predict the probability of harboring *EGFR* mutation by calculation the respective points of three variables (sex, peripheral emphysema, and R-score), with a promising AUC of 0.734. Another promising technique (i.e., liquid biopsy) was consider as an alternative to test *EGFR* mutations. However, the high false-negative rate (30%) need to be further resolved (33).

In patients with NSCLC, the most commonly found *EGFR* mutations are deletions in exon 19 (*Exon19del* in 45%) and in exon 21 (*L858R* in 40%) (2). Both mutations result in activation of the tyrosine kinase domain, and both are associated with sensitivity to the small molecule TKIs. Nevertheless, patients with exon 19 deletion are associated with longer PFS compared to those with L858R mutation after first-line *EGFR*-TKIs (34, 35). In addition, the incidence of T790M mutation, which is associated with acquired resistance to reversible *EGFR*-TKIs (36), might be different between exon 19 deletions and L858R mutations (6), resulting in different treatment scenarios. As a result, furtherly predicting the specific *EGFR* mutation subtypes may be clinically important. Inspired by the satisfied results of predicting *EGFR* mutation status, the potential relations between clinical factors, imaging semantic features, imaging radiomic features, and *EGFR* mutation subtypes were also investigated in our study. Age was the only independent factors that can be used to differentiate different *EGFR* mutation subtypes. No CT features were identified as potential predictors, which is consistent with previous studies (37, 38), indicating the difficult of predicting *EGFR* mutation subtypes through semantic features (relatively low-level). This conclusion encouraged us to investigate whether the radiomic features (relatively high-level) might be competent on this task. Surprisingly, the constructed R-score^*^ was identified as an independent predictor and obtained a good performance with an AUC of 0.708. A radiomics-based nomogram^*^, incorporating age and R-score^*^, achieved a better performance (AUC = 0.757), indicating the efficiency of radiomic features in different medical tasks.

We may conclude that radiomics outperformed the clinical and semantic features in both tasks (see Table 5). The diagnostic benefits may due to the possession of more presentative and discriminative information of radiomics, which could reflect the tumor spatial heterogeneity, tumor microenvironment, as well as tumor gene patterns. Yet, completely interpreting the association between radiomics and the complex biological processes (EGFR mutation status in the current study) remains an intractable challenge. The interpretability warrants further investigation. Please note that one of the implications of our study was to introduce the nomogram, which was easily used in clinical practice and may facilitate the clinical transformation of radiomics researches.

There are several limitations should be noted. First, although the imaging normalization and reproductive analysis can mitigate the influence of radiomic feature variabilities, it cannot make the current study completely immune to the potential confounding variability caused by different CT scanning parameters (39, 40). Paradoxically, studies with homogeneous images must sacrifice the amount of data. How to balance it well is difficult for each radiomics-based research. Second, this was a single-center study and lacked of external validation, indicating the potential data selection bias. Conducting a multi-center study and validating the constructed model in an independent external dataset may not only improve the generalization and robustness of a model efficiently, the models can also substantially benefit from diversified data from different regions, races and countries. Third, the current study narrowly focused on *EGFR* mutation status and subtypes. Constructing a nomogram which could cover all clinical relevant *EGFR* mutation and even other genetic mutations (e.g., *ROS-1, ALK*) may be interesting and worth investigating in the future work.

## Conclusion

Radiomics-based nomogram, incorporating clinical characteristics, CT features and radiomic features, can non-invasively and efficiently predict the *EGFR* mutation status and thus potentially fulfill the ultimate purpose of precision medicine. The methodology is a possible strategy to predict *EGFR* mutation subtypes, providing the support of clinical treatment scenario.

## Data Availability Statement

The datasets generated for this study are available on request to the corresponding author.

## Ethics Statement

This retrospective study was approved by the Institutional Review Board of our institution (No. 20170103), which waived the requirement for patients' informed consent referring to the CIOMS guideline.

## Author Contributions

ML, JL, and WZ: conception and design and provision of study materials or patients. ML, JL, YW, and YH: administrative support. ML, WZ, YS, PG, MT, WM, CL, and LJ: collection and assembly of data. ML, JL, WZ, and YX: data analysis and interpretation. All authors: manuscript writing and final approval of manuscript.

### Conflict of Interest

The authors declare that the research was conducted in the absence of any commercial or financial relationships that could be construed as a potential conflict of interest.

## Abbreviations

*EGFR*: epidermal growth factor receptor
NSCLC: non-small cell lung carcinoma
PFS: progression-free survival
*ALK*: antigen anaplastic lymphoma kinase
*ROS1*: c-ros oncogene 1
TKIs: tyrosine kinase inhibitors.
