# Supplementary material for: The Potential of Radiomics Nomogram in Non-invasively Prediction of Epidermal Growth Factor Receptor Mutation Status and Subtypes in Lung Adenocarcinoma
Source: Front Oncol. 2020 Jan 9;9:1485. doi: 10.3389/fonc.2019.01485 (PMC6962353; doi:10.3389/fonc.2019.01485)
Supplement: Supplementary file 1 [file Table_1.DOCX]

**The formula for calculating the R-score***

R-score*= - 47.931764 - 0.038716 * mean_50_0 - 0.119764 * skewness_0 - 0.042751 * LRE_45_0 - 7.95E-05 * GLN_90_0 + 14.359453 * Homogeneity 0_90_0 - 0.000512 * Contrast 45_90_0 + 9.300583 * Homogeneity 45_90_0 + 11.810171 * Correlation 90_45_0 + 5.793450 * Correlation 135_45_0 - 41.166159 * Homogeneity 135_135_0 - 6.253886 * Homogeneity 0_0_0 + 0.004818 * mean_50_1 + 0.013794 * LRE_90_1 + 23.124440 * SRE_135_1 - 6.45E-05 * GLN_135_1 + 0.941473 * Correlation 0_135_1 + 58.722215 * Homogeneity 45_90_1 + 0.082737 * mean_50_1.5 + 0.001558 * LRE_90_1.5 + 0.000423 * Contrast 45_45_1.5 + 0.639181564 * Entropy 135_135_1.5 + 8.32E-05 *Contrast 0_0_1.5 - 0.047897 * mean_2 + 0.003473 * SD_50_2 + 0.019148 * LRE_90_2 + 4.18E-06 * RLN_0_2.5 -0.000620 * LRE_45_2.5 + 8.043240 * Homogeneity 0_135_2.5 - 4.14E-06 * Contrast 90_90_2.5 - 9.014722 * Correlation 135_90_2.5 -0.000378 * Contrast 0_0_2.5 + 24.59290414 * Homogeneity 0_0_2.5

*Formula caption*: After performing the LASSO method, a radiomics score was calculated for each patient via a linear combination of selected features that were weighted by their respective coefficients. The formula was presented like a logistic regression formula, such as y=*β*_0_ +*β*_1_*x*_1_ +*β*_2_*x*_2_ +*β*_3_*x*_3_ +*β*_4_*x*_4._ For example, the number - 47.931764 indicated the*β*_0_, the number -0.038716 indicated the*β*_1_, * indicated the multiplication sign, and mean_50_0 indicated the *x*_1_. Please note that *x* was presented as feature_σ, feature_ β/α _σ or feature_θ_φ_σ (see **Table S1**). -7.95E-05 or the similar string was presented as scientific notation.

**Table S1**

**Table S1. Extracted imaging features**

| **Gray-level histogram feature** | **Co-occurrence matrix** | **Run lengths matrix** |
| --- | --- | --- |
| skewness_σ | Contrast θ_φ_σ | SRE_α_σ |
| kurtosis_σ | Correlation θ_φ_σ | LRE_α_σ |
| mean_σ | Entropy θ_φ_σ | GLN_α_σ |
| SD_σ | Energy θ_φ_σ | RLN_α_σ |
| mean_ β_σ | Homogeneity θ_φ_σ | RP_α_σ |
| SD_ β _σ |  |  |

Note. σ denotes the filter value, which may be 0, 1.0, 1.5, 2.0 or 2.5.

α, θ, φ denotes the considered direction, which may be 0º, 45º, 90º or 135º.

β denotes the top percentage of the histogram curve, which may be 10%, 25% or 50%.

**Figure S1**

**
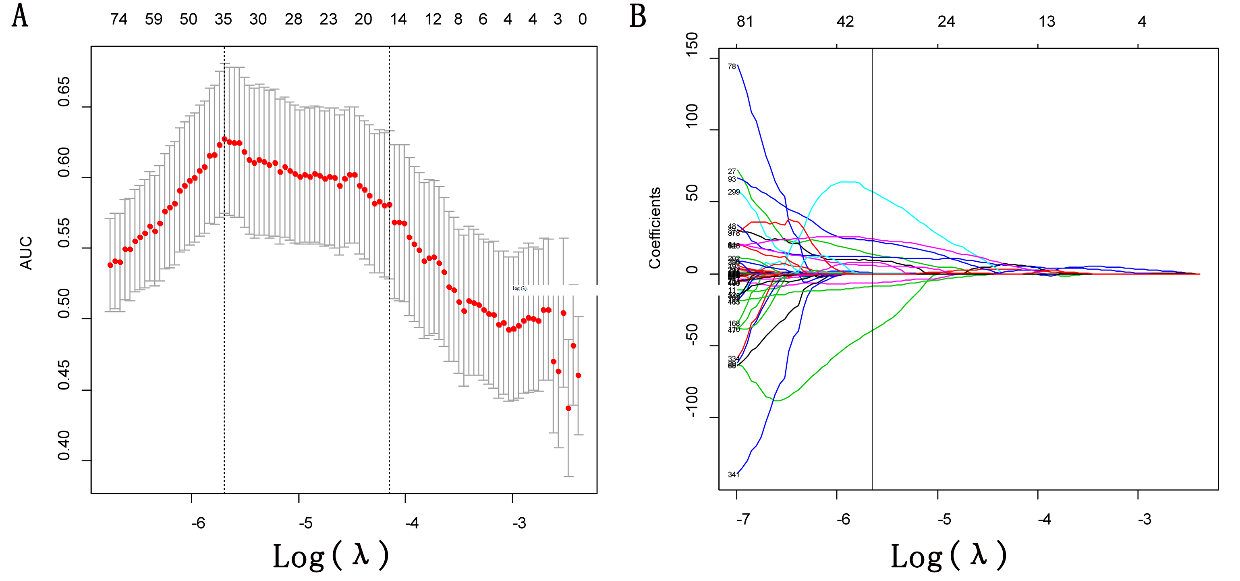
**

Figure S1. Feature selection using the least absolute shrinkage and selection operator (LASSO) binary logistic regression model. (A) Tuning parameter (λ) selection in the LASSO model used 10-fold cross-validation via minimum criteria. The area under the receiver operating characteristic (AUC) curve was plotted versus log (λ). Dotted vertical lines were drawn at the optimal values by using the minimum criteria. (B) LASSO coefficient profiles of the 425 texture features. A coefficient profile plot was produced against the log (λ) sequence. Vertical line was drawn at the value selected using 10-fold cross-validation, where optimal λ (-5.688) resulted in 32 nonzero coefficients.

**Guarantor name：** Ming Li
